# A cross-sectional study on personal values of medical students: the differences from their instructors, their associations with mental wellbeing, and the influences of gender

**DOI:** 10.1186/s12888-024-05695-2

**Published:** 2024-04-12

**Authors:** Romteera Chiencharoenthanakij, Chatchavan Charoenthamruksa, Sansanee Nisu, Krittisak Anuroj

**Affiliations:** 1 Department of Psychiatry, Faculty of Medicine, Srinakharinwirot University, Nakhon Nayok, Thailand; 2 Department of Orthopedics, Faculty of Medicine, Srinakharinwirot University, Nakhon Nayok, Thailand; 3 Department of Psychiatry, Faculty of Medicine, Srinakharinwirot University, 62 Moo 7, Ongkharak subdistrict, Ongkharak district, Nakhon Nayok, 26120 Thailand

**Keywords:** Medical students, Medical instructors, Personal value, Mental health, Thailand

## Abstract

**Background:**

Personal values of Thai medical students have been observed to be diverging from those of their seniors, but the differences remain uncharacterized. Despite its potential association with mental wellbeing, the issue remain unexplored in the population. This study aimed to explore (1) the difference in personal values between medical students and instructors and (2) the association between student’s value adherence to mental well-being and the interaction by gender.

**Methods:**

An online survey was performed in 2022. Participants rated their adherence to five groups of values, namely, Self-Direction, Hedonism, Achievement & Power, Universalism & Benevolence, and Tradition. Participants also rated their mental wellbeing. Comparisons were made between the personal values of students and instructors. The association between the personal values of students and their mental wellbeing and the interaction between values and gender were analyzed in linear regression.

**Results:**

Compared to instructors, students rated higher on Universalism & Benevolence, marginally higher on Hedonism, and lower on Tradition. Students’ ratings on Self-Direction, Universalism & Benevolence, and Tradition predicted better mental wellbeing. Their rating on Hedonism predicted poorer mental wellbeing, the effect of which was marginally stronger in males. Ratings on Achievement & Power marginally predicted poorer mental wellbeing in females.

**Conclusion:**

Difference in personal values between medical students and instructors have been observed. Some of these values hold potentials over student’s mental wellbeing. Curricular and medical school environmental accommodation for the changes in the characters of learners may be necessary to mitigate the adverse effects on their mental wellbeing and foster development of desirable professional characteristics.

## Background

Medical students experience elevated risks of mental health difficulties, such as major depressive disorder, anxiety, sleep difficulties, and suicide [1–9]. Such difficulties could hinder students’ academic performance, create absenteeism, and worsen patient care quality. The difficulties could also deteriorate their social connectivity required for support and healthy personality development. Studies on factors associated with students’ stress present attempts at identifying and correcting predisposing factors toward mental consequences of stress. The current state of literature regarded academic stress as one of the most important contributors [10]. Nevertheless, the ever-changing nature of human society perpetually introduce new challenges into the medical school environment and prompt exploration whether such changes add to the problems of mental health. This could inform the mental health prevention and promotion endeavor in this vulnerable population.

Personal values have been defined as sets of beliefs that pertain to goals and standards that transcend environment-specific norms and can be shaped by generational socioenvironmental circumstances [11]. Some values can coexist, while others, being incompatible, cannot [12]. Multiple values can exist in a person, and manifest behaviors depends on the relative order of importance. Values can be shaped by experience, which accumulates as a person ages, [13] while also being affected by socioenvironmental circumstances of the era. In much of human history, people who coexisted in close time periods, sharing much of their life experiences, tended to possess similar sets of values, the effect of which was termed ‘generation’ [14, 15]. Past society tended to change slowly, and a generation tended to span a long period of time. However, the past century unprecedentedly saw a rapid pace of technological advancements and the coexistence of different generations of people: mathematics teachers who once taught that no one would carry calculators all the time now teach students who carry calculator apps on their phones. Technologies permanently change the way people live. Comfort of living, ease of access to information and communication, and progressively greater reliance on technologies are some notable examples. These have broadened the socioenvironmental experiences between members of society, potentially creating rifts in sets of personal values. Owing to its influence on worldviews and manifest behaviors, relational strains and conflicts between those upholding different sets of values are possible. The magnitude of these conflicts could be pronounced with proximity and demands for exchanges and interrelations, such as in the training of medical professionals. The Thai medical school community have long upheld the values of self-sacrifice and nobleness of medical profession, and displayed conservatism and organizational hierarchy that reflects the broader societal values [16–18]. These, however, do not prevent the societal-wide phenomenon such as shifts in values from reaching the environment. Being involved in medical education, all four authors have noted that the characteristics of medical students have been rapidly changing over the past decade. Altruistic values seem to be in decline, and the gaps in other domains of personal values between medical students and their senior counterparts were widening as well. As the issue of personal values within medical school context has not been systematically characterized in the Thai literature, the authors first sought to characterize them in this study.

The deviation of students’ personal values from the prevailing conservatism in medical school environment, and the resulting differences in values with individuals in their immediate environment such as interns, residents, and instructors, pose risks to students’ mental wellbeing [19]. According to the two-factor model of mental health, low mental wellbeing, aggravated by psychopathology resulting from the interaction of students’ life stress and genetic predisposition, may contribute to the development of mental illness and hinder the attainment of complete mental health [20]. As personal value-derived wellbeing is largely affected by its congruence with societal norms, inconsistencies emerged from cross-cultural studies regarding the association between specific values and wellbeing [21–25]. Research on the influence of values on mental wellbeing within Thai contexts, particularly in medical schools environment, is notably scarce. The conservative nature of Thai society could also drive gender-related differences in values’ influences. This study therefore explored associations between student-held values and mental health and the interaction between gender and values on the association.

## Materials and methods

### Aim

1. Explore the difference in personal values between medical students and instructors.
2. Explore the relationship between medical students’ adherence to values and mental wellbeing and explore the interaction with gender.

### Design, participants, and settings

This study analyzed data from a cross-sectional survey conducted in September 2022 as part of an annual medical instructor’s seminar, part of which concerned the problems of the generation gap in medical schools. The survey used convenience sampling in which the student affairs divisions of all public medical schools in Thailand disseminated the survey and invited affiliated students and medical instructors to participate. Participation was online, anonymous, and voluntary. The survey involved participation from 23 public medical schools, collectively overseeing the training of approximately 15,000 medical students [26]. No personally identifiable data were collected. All respondents were included in this analysis.

### Measures

Demographic data collected included gender, age, religious affiliation, marital status, occupation, affiliation, and the presence of underlying medical and mental diseases. These served as potential covariates for the statistical analysis.

Participants rated the measures of life values on a questionnaire on values created for the purpose of this survey, of which development was based on Schwartz’s theory of values and its derived measure [11, 27, 28]. Adapted for cultural relevance, a total of 15 values were populated, inquiring about the value of one’s (1) physical health, (2) mental health, (3) self-development, (4) private time, (5) leisure activities, (6) engaging activities, (7) occupational security, (8) occupational reputation, (9) salary, (10) family, (11) friends, (12) social tolerance, (13) contribution to one’s own institution, (14) contribution to the world, and (15) religious practices. Adherence to each value was queried in three questions, namely, subjective perception of its importance (which is the main issue queried in measure derived from Schwartz’s theory), reluctance to forego it in favor of other values, and effort being put (or already put) to adopt the value [28]. The latter two sets of questions, being relatively behaviorally-focused, were expanded to address norm conformity of the first sets of perception-based items. Each item was phrased as a statement such as “I am putting effort (or have put effort) to keep myself healthy”, and respondents rated their agreement on a 5-point Likert scale ranging from totally disagree to totally agree. The sum of the three items for each value, which ranged from − 6 (total disregard) to 6 (total endorsement), with 0 denoting indifference, was entered into the statistical analysis. The measure was content validated in two consecutive meetings of the conference subcommittee, from which minor adjustments were made, and underwent face validity testing in healthy volunteers.

Mental wellbeing, the variable outcome of interest, was viewed from the outlook of positive psychology which defined wellbeing from perspectives of positive emotion, engagement, relationship, meaning making, and accomplishment. The employed rating scale, PERMA profiler, was built upon the concept by Butler & Kern [29, 30]. The subscales on positive emotion, meaning making, and accomplishments were employed in in this survey. (The other two subscales were omitted owing to speculation that they could be spuriously affected by the scarcity of free time among medical students to socialize and engage in their activities of interest.) Each item was rated on a 10-point Likert scale. The sum of these items was entered as the dependent variable, mental wellbeing. The employed items had an internal consistency statistics (Cronbach’s alpha) of 0.85.

### Sample size

The sample size was determined prior to the survey using G*Power version 3.1.9.7 [31, 32]. The first objective was analyzed using independent t tests. Specifying alpha = 0.05, power = 0.95, and presumed medium effect size, a sample size of 105 was required in each group. The second objective was analyzed using a multiple regression analysis. Specifying alpha = 0.05, power = 0.95, presumed eight variables, and presumed medium effect sizes of the examined values, a total sample size of 160 was required.

### Statistical analysis

Statistical analyses were performed using IBM SPSS Statistics for Windows version 27 and STATA. The measures of values underwent principal axis factoring with Oblimin rotation to validate its construct. The number of factors extracted were based on factors possessing eigenvalues greater than 1. Factor loadings were considered significant if they exceeded the threshold of 0.3. Naming of the factors were contingent upon the loaded items according to categorization in Schwartz’s theory values [11, 27]. The sum of ratings in each category was entered into the subsequent statistical analyses. Distribution of ratings on values were examined in histograms. Differences in values between students and instructors were examined with independent t tests for normally distributed values, and with Mann-Whitney’s U tests for values violating the normality assumption. The association between values and mental wellbeing among medical students was examined with the main effect model of linear regression. Interaction terms between gender and each value were then added to the model. Subsequently, main effect regression models stratified by gender were constructed to illustrate the difference in B coefficients between genders. Assumptions of independence of errors, normal distribution of residuals, homoscedasticity, and linearity were tested. Restricted cubic spline was used if any covariates violated linearity assumptions. The variable selection method used was backward removal.

## Result

### Characteristics of the respondents

There were 666 respondents to the survey, and their demographic data are presented in Table 1. Females constituted the majority of the respondents. Most were Buddhist. Medical students outnumbered instructors by four to one. Most of the respondents had no underlying diseases. Among those who reported underlying medical diseases, the most common were allergic rhinitis/conjunctivitis (*n* = 55), followed by thyroid disease (*n* = 5), G6PD deficiency (*n* = 4), heart disease (*n* = 3), gastritis (*n* = 3), thalassemia (*n* = 2), hepatitis B carrier (*n* = 2), pes planus (*n* = 1), spondylolisthesis (*n* = 1), endometriosis (*n* = 1), and polycystic ovary syndrome (*n* = 1). Underlying mental disorders were rare and included major depressive disorder (*n* = 4), bipolar disorder (*n* = 1), and attention deficit hyperactivity disorder (*n* = 1). The survey did not contain any missing data.

**Table 1:** Demographic data, rating on values, and mental wellbeing rating of the participants

Variable	Students (n = 520)	Instructors (n = 146)
	N (%) or mean (SD)	N (%) or mean (SD)
Age	20.0 (2.3)	40.7 (9.9)
Gender		
- Female - Male - Gender minorities	305 (58.7) 192 (36.9) 23 (4.4)	86 (58.9) 58 (39.7) 2 (1.4)
Status		
- Single - Married - Other	519 (99.8) 1 (0.2) 0 (0)	75 (51.4) 63 (43.2) 8 (5.5)
Religion		
- Buddhism - Others (Christian, Islam, Sikh, and Areligious)	465 (89.4) 55 (10.6)	135 (92.5) 11 (7.5)
Affiliation		
- Metropolitan medical school - Regional medical school	242 (46.5) 278 (53.5)	108 (74) 38 (26)
Underlying physical illness		
- Absent	456 (87.7)	120 (82.2)
- Present	64 (12.3)	26 (17.8)
Underlying mental illness		
- Absent - Present	515 (99.0) 5 (1.0)	144 (98.6) 2 (1.4)

### Personal value measure

From the principal axis factoring of measures on values, the Kaiser-Meyer-Olkin (KMO) measure of sampling adequacy was computed and yielded a value of 0.83, a meritorious level. Bartlett’s test of sphericity revealed significant deviation from sphericity (χ² = 4121.80, df = 105, *p* < 0.01). A total of five factors were extracted based on eigenvalues exceeding 1.0, explaining 57.7% of variance. The five extracted factors corresponded to (1) Self-Direction (included items on physical health, mental health, and self-development), (2) Hedonism (private time, leisure activities, and engaging activities), (3) Achievement & Power (occupational security, occupational reputation, and salary), (4) Universalism & Benevolence (contribution to own institution, contribution to the world, social tolerance, family, and friends), and (5) Tradition (religious practices.) These value categories are capitalized so as not to be confused with common words in the following texts.

### The differences in personal values

The ratings on Self-Direction exhibited a left-skewed distribution among both students and instructors. No statistically significant difference between the groups was observed in Mann-Whitney’s U test (U = 38,087.5, significance = 0.95), of which the significance level was similar to mean comparison using t test (t = -0.42, *p* = 0.97).

**Fig. 1:**
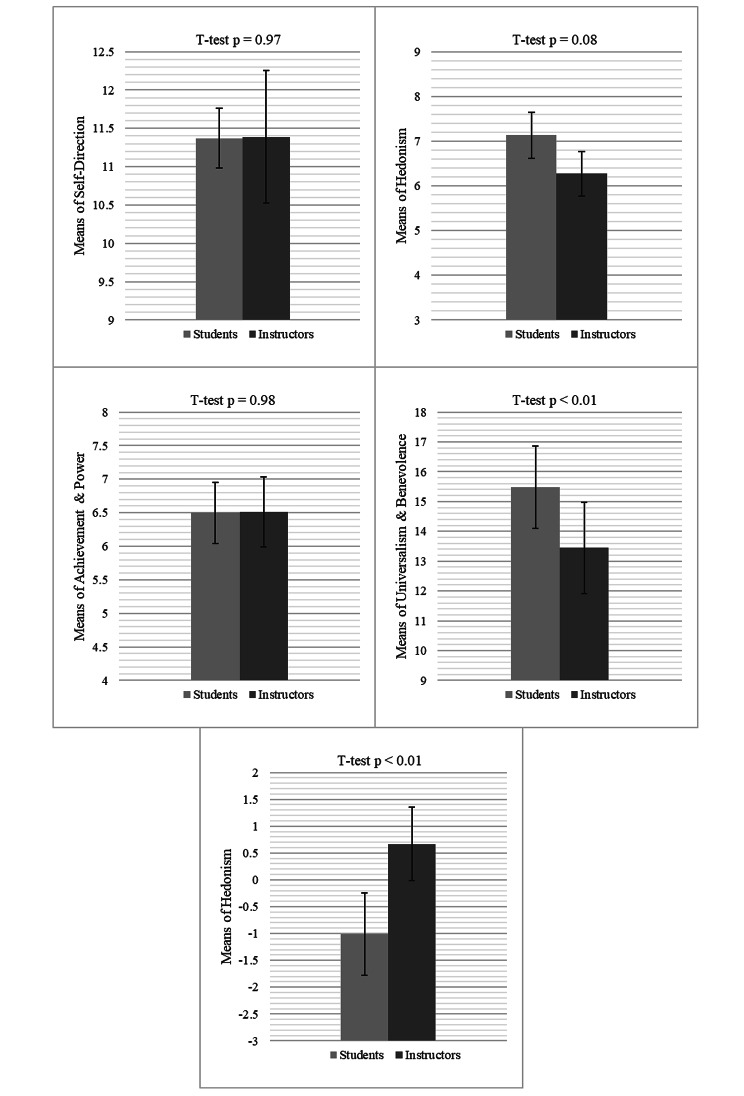
Mean plots of ratings on five life value categories among medical students and instructors

The remaining groups of values conformed with normality assumptions of the independent t test. The analyses showed that students rated higher on values in Universalism & Benevolence (t = 3.01, *p* < 0.01), marginally higher on Hedonism (t = 1.74, *p* = 0.08), and lower on Tradition (t = -6.38, *p* < 0.01). There were no significant differences in the ratings on Achievement & Power (t = -0.02, *p* = 0.98). The mean plots are shown in Fig. 1.

### Personal values and mental wellbeing

Linear regression was utilized to examine the association between the ratings on personal values and mental wellbeing among the 520 medical students. The evaluation of regression assumptions revealed that the variable “age” did not conform to the linearity assumption, most likely due to its relationship with different stress levels from different academic years. Therefore, restricted cubic splining was performed. From the regression model adjusted for splined age, gender, marital status, religion, presence of underlying medical disease, and presence of underlying mental disease, it was found that ratings on Self-Direction, Universalism & Benevolence, and Tradition predicted higher mental wellbeing (b = 0.23, *p* < 0.01; b = 0.12, *p* < 0.01; and b = 0.24, *p* < 0.01, respectively), while the rating on Hedonism predicted lower mental wellbeing (b = -0.16, *p* < 0.01). Parameter estimates from the main effect model are presented in Table 2. The regression model met the regression assumptions of independence of errors (Durbin-Watson statistics = 1.91), normal distribution of residuals (visualized from histogram and Q‒Q plot), and homoscedasticity (visualized from scatterplot).

**Table 2:** Parameter estimates from the linear regression model with mental wellbeing as the dependent variable. The results from the main effect models analyzing all students and stratified analyses by gender are presented in the upper section. Interaction analysis between value and gender is presented in the lower section

Variable	All students		Female		Male		Gender minorities		
	B (95% CI)	*P* value	B (95% CI)	*P* value	B (95% CI)	*P* value	B (95% CI)	*P* value	
**Main effect model**									
Self-Direction	0.23 (0.11–0.33)	< 0.01	0.19 (0.05–0.34)	0.01	0.25 (0.06–0.44)	0.01	0.15 (0.31–0.61)	0.52	
Hedonism	-0.16 (-0.25– -0.08)	< 0.01	-0.11 (-0.22–0.00)	0.05	-0.24 (-0.38–0.08)	< 0.01	-0.26 (-0.62–0.10)	0.15	
Achievement & Power	-0.03 (-0.13–0.05)	0.50	-0.11 (-0.22–0.00)	0.06	0.00 (− 0.15–0.15)	0.97	0.27 (-0.10–0.63)	0.15	
Universalism & Benevolence	0.12 (0.06–0.19)	< 0.01	0.13 (0.04–0.22)	< 0.01	0.13 (0.02–0.24)	0.02	0.16 (-0.06–0.38)	0.16	
Tradition	0.24 (0.09–0.37)	< 0.01	0.28 (0.11–0.46)	< 0.01	0.18 (-0.10–0.46)	0.21	0.16 (-0.43–0.75)	0.60	
**Interaction model**									
Self-Direction * Gender	Not significant								
Hedonism * Male gender	-0.14 (-0.30–0.04)	0.12							
Hedonism * Gender minorities	-0.18 (-0.53–0.18)	0.33							
Achievement & Power * Male gender	0.12 (-0.05–0.30)	0.17							
Achievement & Power * Gender minorities	0.44 (0.10–0.78)	0.01							
Universalism & Benevolence * Gender	Not significant								
Tradition * Gender	Not significant								

95%CI: 95% confidence interval of the regression coefficient

Main effect models were adjusted for restricted-cubic-splined age, gender, marital status, religion, presence of medical underlying disease, and presence of mental underlying disease.

Coefficients of determination (R^2^) were 0.42 for the main effect model and 0.43 for the interaction model.

### Interaction between personal values and gender

Interaction terms between values and genders were subsequently entered into the regression model. With female as the reference category, a trend toward a negative interaction between male gender and Hedonism was found (B = -0.15, *p* = 0.1). The magnitude of Hedonism was apparently greater among males in the regression model stratified by gender (B = -0.24, *p* < 0.01, compared to B = -0.11, *p* = 0.05 for females). A trend toward positive interaction between Achievement & Power with male gender and a significant positive interaction with gender minorities were also noted. From the stratified analysis, females had a marginally significant negative parameter estimate for the value (B = -0.11, *p* = 0.06) in contrast to other genders possessing no statistically significant coefficient. Details on the parameter estimates of the interaction models and stratified regression models are displayed in Table 2.

## Discussion

This study was the first to examine the value systems of medical students and instructors in Thailand. The analysis has confirmed the existence of a difference in personal values between students and instructors. The difference can be viewed as a product of generation-wide societal phenomena on one hand, and of accumulated personal experience and personality development through progression of life cycle on the other. Students rated significantly higher on Universalism & Benevolence, which included values on family, friends, social tolerance, contribution to own institution, and contribution to the world. This implies that students require more time and depth in social life than their seniors. The higher inclination toward friends and intimate significant others could be the manifestation of psychosocial developmental stage [33]. Attachment, reliance, and subsequent valuing of family could have also been affected by generational changes in parenting style, which not only included an increase in democratic elements, but also greater degrees of overprotection and overindulgence [34, 35]. The practices nonetheless offered closer relationship opportunities for children when compared to the authoritarian style of the past. One explanation of the rise in overprotective and overindulgent behaviors is that these parents are from the recently expanded middle class [36]. Their life struggle, combined with absorbed Western values, caused their parenting practices to deviate from those of the previous generation [37–39]. These, combined with the ease of living from technological advancements, could have produced an increase in inclination toward hedonistic ways of life [40]. Early exposure and ease of access to mobile entertainment can also prime them toward hedonism [41]. All of these could be intensified by students’ psychosocial developmental stages of late adolescence and early adulthood, in which the foci are directed relatively inward to the self [33]. Hedonism is a value incompatible with altruistic traits owing to its self-centered aspects [12]. Despite the observed difference being marginal, the incompatibility holds the potential to hinder meaning making in the profession and create relationship strains with seniors and instructors, contributing to disruptions of student’s mental wellbeing as well. Hence, future studies verifying the trend of change in hedonism in the context is still warranted.

The lower rating on religious values among students could be a reflection of the increase in religiosity in Thai society [34, 42]. This could be a result of the rise of social consciousness following easy and widespread access to media, disillusionment with current religious institutions, and incompatibility with modern consumerist ways of life [43–47]. Rise in irreligiosity is also an evolving trend in Western countries as well [48]. 

Nevertheless, ratings on the other two sets of values, Self-Direction and Achievement & Power, were similar between the groups. Both students and instructors valued the ability to be in charge of their lives and valued career-related achievements all the same. Though the specific foci may differ by developmental stages, basic psychological desires toward self-realization [49] and exertion of control over one’s immediate environment [50] appear to operate regardless of age.

Studies in Western societies have noted that values related to personal growth and self-transcendence were associated with positive affectivity and wellbeing, but replication in Asia has been scarce [51]. This study analyzed the effect of endorsing different values on the mental wellbeing of medical students. Consistent with a previous study in the West, the measure on Self-Direction predicted better mental wellbeing [52]. This category of value implies the ability to redirect attention toward one’s well-being in the face of adversity and results in better self-care behaviors. The ratings on Universalism & Benevolence also predicted better mental health. The value partly concerned the person’s investment in social circles and subsequent supports, which are protective against mental difficulties [53]. The value also concerned institutional and world contributions, which are natural elements of healthcare work and can serve as a guide to students’ life directions. Consistent with the concept of Logotherapy, by seeing meaning in their routine studies and patient care responsibilities, students become buffered from the adverse mental effects of medical school lives [54]. In contrast with speculations from liberal perspectives that view traditions as restricting, the present study observed similar findings to another study in a conservative society that the valuing Tradition predicts better mental wellbeing [51, 55]. Religion, although in decline, can exert protective effects on one’s mental wellbeing, such as by providing mental respite in prayer and meditation [42]. It also adds to meaning where there is nothing to be seen. In the context of Buddhism, adverse events in life are commonly attributed to “bad karma” committed in past lives. Following such philosophical consciousness, people let things go so as not to perpetuate karma.

Valuing Achievement & Power is associated with self-expansion and growth and, as discussed, has been linked with positive affectivity. In contrast, the results from the whole sample did not reveal an association between this value and mental wellbeing. It is possible that the stressful learning environment and intensive norm-referenced assessment deprived students of the sense of academic accomplishment. As Thai medical students typically had backgrounds as accomplished high schoolers and were primed to the glory of academic achievement, the interaction between this value and the inevitable deprivation of success could serve to injure their self-esteem. Moreover, the result from stratified analysis showed that adherence to this value appeared to predict poorer mental wellbeing exclusively in females. Interaction analysis between this value with gender, however, showed only a trend toward significance, suggesting a potentially small effect size for the interaction that is underpowered to detect with the current sample size. Given the potential influences from gender differences in achievement perception, trait emotionality, and societal pressure on female students’ wellbeing, the observed trend necessitates further exploration to inform the creation of inclusive and equity-focused educational environment [56–58]. 

Hedonism, as studied in the Western context, has been viewed as a part of modern values and has been positively associated with mental health [51]. However, the present study observed the opposite. The value, which may be on its early course of rising, pertains to the inclination toward pleasurable activities and pastime. Hedonism is philosophically antagonistic to Altruism, potentially impeding students’ meaning making of the hardship of healthcare work. Students who endorse highly in Hedonistic lifestyle could be frustrated by the limiting nature of medical school environment. A trend toward magnification of the negative effect was noted among male students; however, the observed effect size could have been small, potentially contributing to the lack of statistical significance. The added vulnerability, if present, could be attributed to societal expectations of emotion regulation behaviors. Like many other cultures, social norms dictate that Thai males display little depressive emotion both in public and close circles, in contrast to females, who typically can express sadness while confiding in peers or family [59]. Reliance on hedonistic behaviors can be seen as one of the few available outlets and the deprivation of such coping consequently affects males more. Another possibility lies in the engagement in harmful hedonistic behaviors such as alcohol, gambling, or substance misuse, which can be seen more frequently in males and adversely affect their mental health in the long run [60]. Hedonism can also be viewed negatively be senior members of the medical community, who may derogate the trait as indolence or intolerance, and feel compelled against forming workable relationship with these younger members of the community. As the trait is yet to pervade into normalcy of the community, seeking middle ground between adaptation and acceptance appears to be a viable option.

### Implications

Owing to the dynamic nature of human society, differences in values among members of the medical community appear inevitable. For now, some differences such as in the Hedonism values may still be subtle, the study of which would require sample size large enough to achieve sufficient statistical power. The shifts in personal values would subsequently alter manifest behaviors and attributes of medical students and future candidates. Recognition must be made that baseline attributes of candidates have started to shift, and medical education curriculum needs to adapt to address the issue. Whether to accommodate or assimilate is still the question. The authors believe that the judgement should be made on whether the values ultimately affect the desirable characteristics of medical students and professionals. Along with this, the definition of what is considered desirable should evolve as well, incorporating views from younger members of the medical communities and the expectations from the wider society. Indeed, some changes brought about by the younger members such as the directness in communication and desire for transparency could be for the better of the community [61]. Supposed that the rise in Hedonism is confirmed in subsequent studies, medical school may opt to accommodate the trait by allowing for more forgiving study and work hours as long as the desired threshold of knowledge, skills, and attitude can be reached. However, should any traits appear to deteriorate the student’s desirable characteristics or mental wellbeing, the decision whether to intervene must be contemplated by the curriculum designers, who should be informed by empirical evidence and engagement of stakeholders, including students themselves. As the manifestation of two competing values into behaviors can be affected by its personally assigned order of importance, achievement of desirable professional characteristics and behaviors could partially be met through interventions targeting student’s values within the process of medical education. The breadths and scopes of educational processes already in place, such as feedback, reflection, and clinical exposure, can be adjusted to cover the issues of values as well.

As there may exist gender-related differences in the manifestations and consequences of values, actions should be taken to ensure that perspectives are heard from diverse and representative cast before relevant administrative decisions are made.

### Limitations

The observed difference in values cannot be attributed to the effect of generations alone, as respondents were from various psychosocial developmental stages. The cross-sectional nature of the survey limits the strength of the conclusions on directionality. Relatively small numbers of gender minority participants were recruited, and whether their adherence to values affects mental wellbeing differently from other genders still require further studies. For medical schools in different cultures, the exact findings may possess limited generalizability but should nonetheless prompt investigations and actions. Furthermore, as the long-term effects of values, and changes thereof, among medical students and new professionals are not known, longitudinal studies, such as those exploring graduate’s adjustment to the professions or the long-term mental wellbeing outcome, could invaluably inform practices in the field of medical education.

## Conclusion

Medical students possess different sets of values compared to their instructors, particularly values related to Universalism & Benevolence, Hedonism, and Traditions. Furthermore, ratings on values related to Self-Direction, Universalism & Benevolence, and Tradition predicted better mental wellbeing, while the rating on Hedonism predicted poorer mental wellbeing, the effect of which was stronger in males. Among females, ratings on Achievement & Power showed a trend toward predicting poorer mental wellbeing. Adaptations of the medical school curriculum to reinforce positive traits and accommodations’ generational demands are discussed.

## Data Availability

The data that support the findings of this study are available from the student affairs division of the faculty of medicine, Srinakharinwirot University, but restrictions apply to the availability of these data, which were used under license for the current study, and so are not publicly available. Data are however available from the corresponding author upon reasonable request (email: thymelancille@gmail.com) and with permission of the organization in possession of the data (contact address: Student affairs division, Faculty of Medicine Building, Srinakharinwirot University, 62 Moo 7, Ongkharak subdistrict, Ongkharak district, Nakhon Nayok province, Thailand. Postal code: 26120).

## Declarations

### Authors’ information

RC and KA are affiliated with the department of psychiatry, faculty of medicine, Srinakharinwirot University, of which SN is the departmental head. KA and SN are also affiliated with the institution’s student affairs division, of which CC was the responsible deputy dean at the time the survey was conducted.
